# Early diagnosis of streptococcus cristatus in blood culture-negative infective endocarditis by capture-based metagenomic next-generation sequencing: a case report

**DOI:** 10.3389/fcvm.2025.1604687

**Published:** 2025-08-07

**Authors:** Dong Wang, Hui Wang, Dan Zhang, Xinsheng Yan

**Affiliations:** Department of Clinical Laboratory, Wuhan Asia General Hospital, Wuhan Asia General Hospital Affiliated to Wuhan University of Science and Technology, Wuhan, China

**Keywords:** infective endocarditis, *Streptococcus cristatus*, capture-based metagenomic nextgeneration sequencing, valve vegetations, case report

## Abstract

**Background:**

Infective endocarditis (IE) is a life-threatening infectious cardiac condition characterized by therapeutic complexity and high mortality rates, for which precise pathogen identification is critical to guide accurate treatment. Although this disease is frequently caused by commensal microorganisms of the oral flora, including *Streptococcus cristatus* (*S. cristatus*); however, *S. cristatus* is not a common pathogen associated with IE.

**Case presentation:**

A 59-year-old male patient was admitted to our intensive care unit due to chest tightness and shortness of breath persisting for 10 days, with symptoms worsening over the last 6 h, including dyspnea and an inability to lie down. After the patient was admitted to the hospital for comprehensive examinations, a preliminary clinical diagnosis of IE, aortic valve vegetation formation, acute non-ST-segment elevation myocardial infarction, and heart failure was established. The patient had negative preoperative blood culture results and received empiric therapy with moxifloxacin combined with piperacillin-tazobactam for infection control, subsequently undergoing cardiac surgery. Intraoperatively obtained valve vegetations were sent for pathological testing, tissue bacterial culture, and capture-based mNGS (metagenomic next-generation sequencing) testing. The capture-based mNGS results for the vegetation was returned as *S. cristatus* within 24 h, with 250,119 sequences detected and 54.56% coverage, which facilitated the rapid identification of the pathogenic microorganism of IE in the early stage. The tissue culture result of the vegetation was returned on the 5th day of delivery, confirming the presence of *S. cristatus*. The patient was successfully discharged after comprehensive treatment and returned to the hospital 3 weeks post-discharge for a follow-up examination, which suggested a good recovery.

**Conclusions:**

This case highlights a rare instance of *S. cristatus* endocarditis, which was ultimately confirmed at an early stage through capture-based mNGS performed on valvular vegetation. This suggests that for postoperative patients with persistent infection and blood culture-negative IE, valvular capture-based mNGS serves as a rapid and efficient diagnostic tool to expedite pathogen identification and guide targeted antimicrobial therapy.

## Introduction

Infective endocarditis (IE) is an inflammation of the heart valves and/or the lining of the heart caused by infection with bacteria, fungi, or other pathogenic microorganisms, including viruses and chlamydia (1). This condition is associated with a high mortality rate and serious complications (2). The modified Duke criteria remain the primary standard for IE diagnosis, integrating clinical features, imaging findings (e.g., echocardiography), and laboratory tests such as histopathology, microbial culture, serology, and PCR-based assays (1). Positive blood cultures remain the gold standard for diagnosing infective endocarditis (IE) and provide viable bacteria for both identification and susceptibility testing (2). However, they may fail to detect pathogens due to clinical factors, such as the preoperative administration of antibiotics to patients. Additionally, fastidious bacteria, intracellular pathogens, fungal infections, and low microbial loads can also contribute to blood culture-negative endocarditis (BCNIE) (2). Blood culture-negative infective endocarditis accounts for 10%–35% of all IE cases (3–6). The absence of etiologic confirmation poses significant challenges to early diagnosis and targeted therapy. Furthermore, accurate etiologic identification is critical for optimizing antimicrobial therapy and improving clinical outcomes in IE. Recently, metagenomic next-generation sequencing (mNGS) has emerged as a non-culture-based molecular diagnostic tool for the pathogenic diagnosis of infectious diseases, capable of detecting a wide range of potential pathogens in clinical samples (7, 8). However, there are few studies on the application of capture-based mNGS in the pathogenetic diagnosis of IE vegetations (9, 10). In this article, we present a case of blood culture-negative *Streptococcus cristatus* (*S. cristatus*) native-valve endocarditis that was rapidly diagnosed through capture-based mNGS analysis of valvular vegetations. This report aims to provide diagnostic insights for the etiological identification of *S. cristatus* endocarditis.

## Case presentation

**Figure 1:**
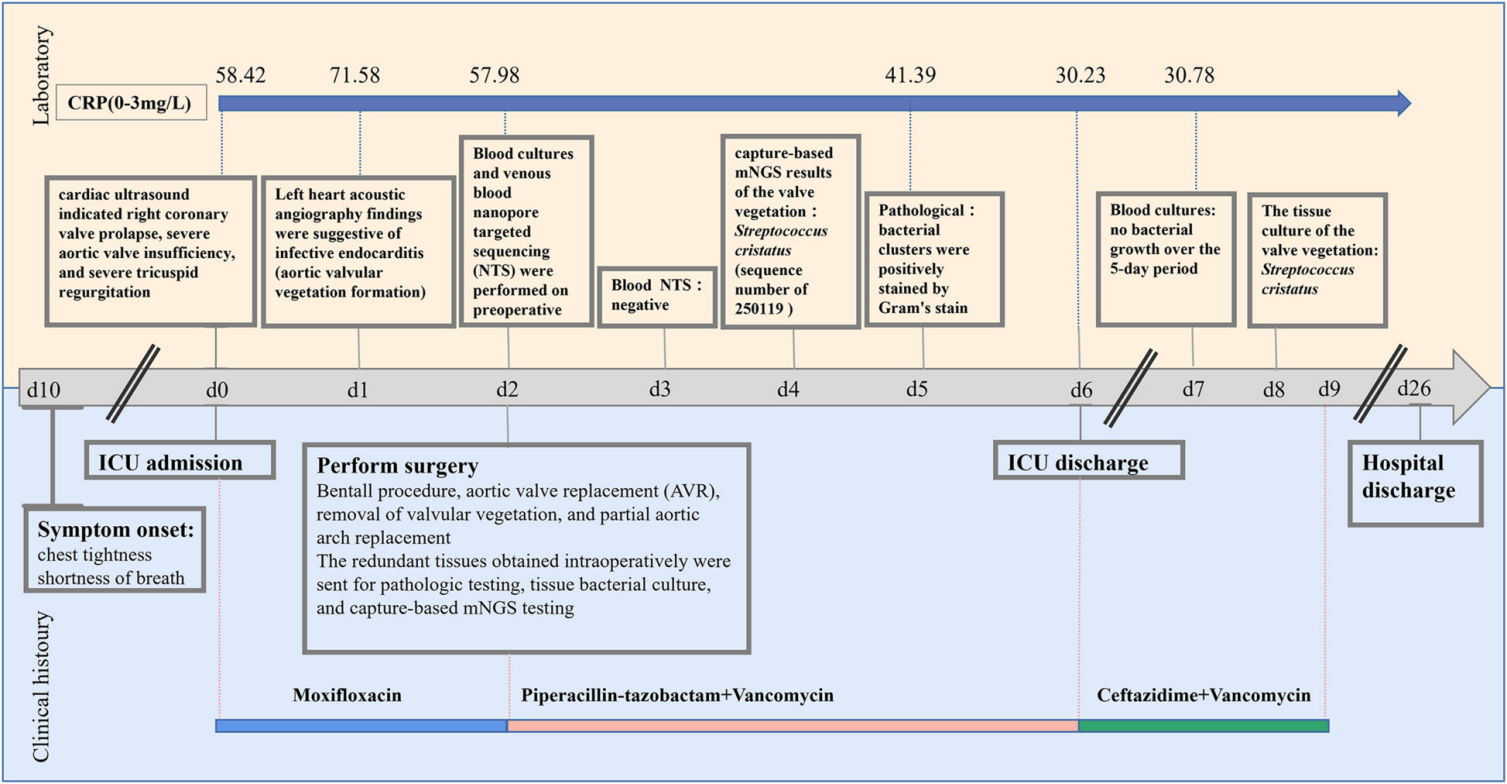
Timeline of patient's clinical management progression, including timing of clinical histoury, diagnostic testing and antimicrobial therapy. ICU admission is used as the day 0 reference point.

A 59-year-old male patient was admitted to our intensive care unit (ICU) on January, 2025, due to chest tightness and shortness of breath persisting for 10 days, with symptoms worsening over the last 6 h, including dyspnea and an inability to lie down (Figure 1). Upon admission, examination revealed a body temperature of 36.5°C, heart rate of 122 beats per minute, respiration rate of 30 breaths per minute, blood pressure of 102/49 mmHg, and an SPO_2_ of 80% while receiving 6 L/min of oxygen via mask. Auscultation of both lungs revealed coarse respiratory sounds, along with the presence of dry and wet rales. A diastolic sighing murmur was noted at the right edge of the sternum in the second intercostal space, while the remainder of the vital signs remained normal. On the day of admission, cardiac ultrasound indicated right coronary valve prolapse, severe aortic valve insufficiency, and severe tricuspid regurgitation. The electrocardiogram demonstrated sinus tachycardia, poor R-wave progression in the anterior wall leads, and ST segment depression in leads V5–6. Chest CT revealed exudative infection in both lungs. Laboratory tests revealed the following results: SARS-CoV-2 nucleic acid was negative, while influenza A virus nucleic acid was positive and influenza B virus nucleic acid was negative. The NT-proBNP level was measured at 20092.0 pg/ml (0.0–125.0 pg/ml). Electrolyte levels included blood potassium at 4.58 mmol/L (3.5–5.3 mmol/L), blood sodium at 138.2 mmol/L (137.0–147.0 mmol/L), blood chloride at 109.1 mmol/L (90.0–111.0 mmol/L), and blood calcium at 2.15 mmol/L (2.11–2.52 mmol/L). Additionally, high-sensitivity troponin T was recorded at 1,755.00 ng/L (0.00–14.00 ng/L), lactate at 3.06 mmol/L (0.5–2.2 mmol/L), interleukin 6 at 65.20 pg/ml (0.00–7.00 pg/ml), and procalcitonin at 0.464 ng/ml (0.00–0.06 ng/ml). The white blood cell count was 12.23 × 10^9^/L (3.50–9.50 × 10^9^/L), with a neutrophil percentage of 88.0% (40.0%–75.0%), and hemoglobin level at 135 g/L (130.0–175.0 g/L). The patient was admitted with initial diagnoses of heart valve disease, acute non-ST-segment elevation myocardial infarction, and pulmonary infection.

Based on the date of ICU admission (Day 0). On day 1, contrast-enhanced transthoracic echocardiography (TTE) with left heart contrast echocardiography revealed findings suggestive of infective endocarditis, including aortic valvular vegetation formation (attached to the right coronary cusp, ∼1.3 × 0.6 cm), prolapse of the right coronary cusp with severe aortic regurgitation, aneurysmal dilatation of the ascending aorta (inner diameter 5.7 cm; aortic sinus 4.4 cm; sinotubular junction 3.9 cm), and mild regurgitation of the mitral and tricuspid valves (Table 1). Currently, the diagnosis of infective endocarditis, aortic valve growth, acute non-ST-segment elevation myocardial infarction, and heart failure is being considered. From Day 0 to Day 2, empirical antibiotic therapy was initiated with moxifloxacin (0.4 g, administered via infusion pump daily), and a consultation with the cardiothoracic surgery department was requested to evaluate the indications for surgery. On Day 2, a combination of moxifloxacin and piperacillin-tazobactam (4.5 g, administered via infusion pump every 6 h) was administered for a brief period, just prior to the surgery.

**Table 1:** The data of left heart contrast echocardiography and transthoracic echocardiogram.

Detection method	Aneurysmal dilation of the ascending aorta			Aortic valvular vegetation (cm)
	Inner diameter (cm)	Sinus region (cm)	Sino-glomerular junction (cm)	
Left heart contrast echocardiography	5.7	4.4	3.9	1.3 × 0.6
Transthoracic echocardiogram	6.3	4.2	3.9	1.5 × 1.0

On day 2, the cranial CT scan of the patient revealed no significant abnormalities. The CTA of the head, carotid artery, great vessels, coronary artery, heart, chest, and abdomen indicated the following: atherosclerosis of the carotid and cerebral arteries; the aorta appeared normal; aneurysmal dilation of the ascending aorta; coronary arteries showed no plaque or stenosis; a myocardial bridge was observed in the middle of the left anterior descending artery; interstitial infections were present in both lungs; a moderate amount of effusion was noted in the right thorax, with a small amount in the left thorax. The patient was administered symptomatic treatments, including anti-infective (moxifloxacin and piperacillin-tazobactam), antiviral, expectorants, cardiotonics, diuretics, vasodilators, and anticoagulants. Following a consultation with the cardiothoracic surgery team, the patient and their family consented to proceed with surgery for Bentall procedure, aortic valve replacement (AVR), removal of valvular vegetation, and partial aortic arch replacement. Blood cultures and venous blood nanopore targeted sequencing (NTS) tests were conducted on day 2. The NTS results returned negative on day 3, and the blood cultures were reported on day 7, showing no bacterial growth over the 5-day period.

On the afternoon of day 2, the patient underwent the Bentall procedure, partial aortic arch replacement with a prosthetic graft, tricuspid valvuloplasty, removal of aortic valve vegetations, and intraoperative temporary pacemaker implantation. During the surgery, a valved conduit comprising a 25 mm Bioroot Medical aortic valve and a 30 mm branched artificial graft was implanted. The patient was transferred to the ICU postoperatively for routine postoperative monitoring and life support therapy.

**Figure 2:**
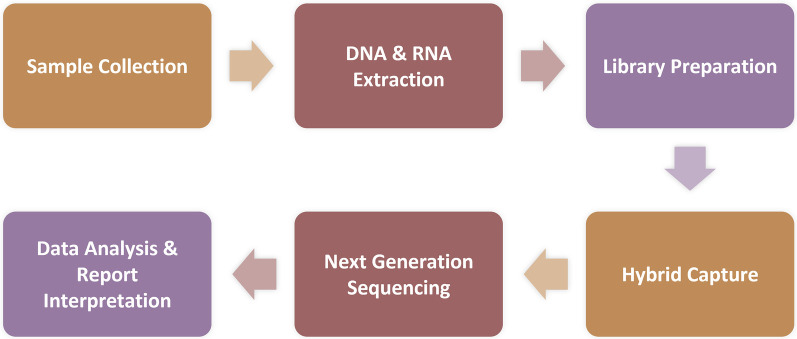
Protocol for capture-based mNGS assay. **Sample Processing and Nucleic Acid Extraction:** Blood samples (3 ml each) were centrifuged at 1,600 × rcf for 10 min at 4°C. Nucleic acid was extracted from 300 μl of the resulting plasma using the MasterPure DNA & RNA Extraction Kit (KingCreate) according to the manufacturer's instructions. The extracted nucleic acid was then used for library construction; **Library Construction, Enrichment, and Sequencing:** Following RNA conversion to complementary DNA (cDNA), libraries were constructed through fragmentation, end repair, adapter ligation, and PCR amplification. After library generation, eight uniquely barcoded libraries were pooled and hybridized with specific biotinylated probes for 2 h using the MetaCAP Pathogen Capture Metagenomic Assay Kit (KingCreate), according to the manufacturer's protocol. This captured library pool was then sequenced on an Illumina MiniSeq platform.

**Figure 3:**
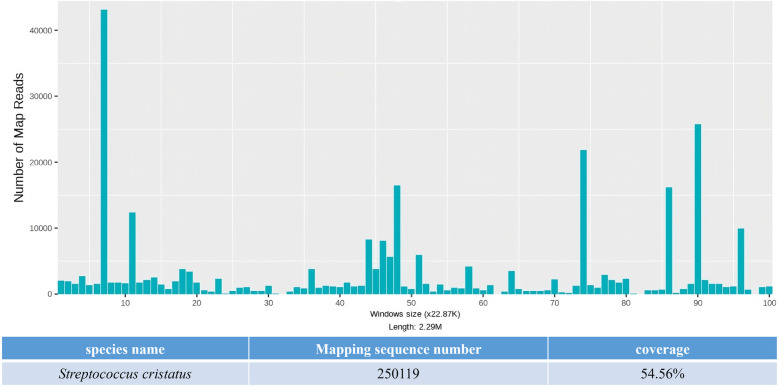
The capture-based mNGS results of pathogens detected in aortic valve vegetation.

**Figure 4:**
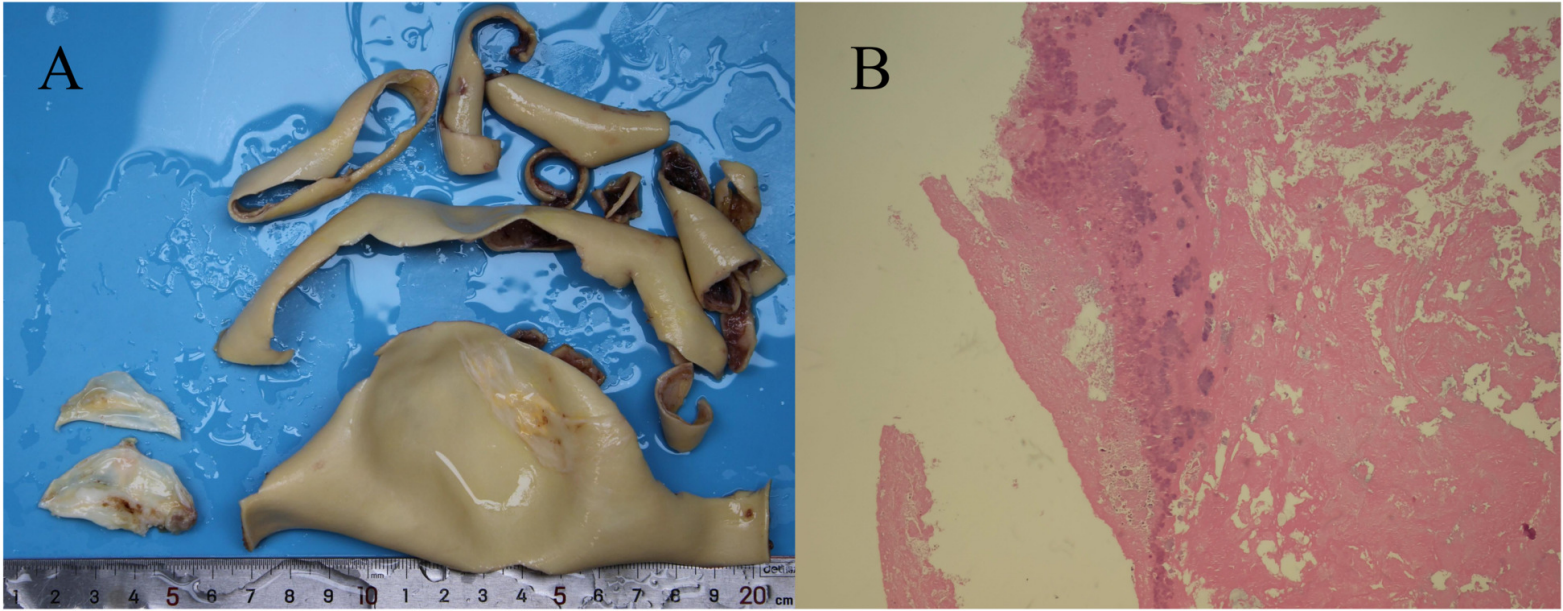
Aortic valve vegetation and pathological analysis. **(A)** A grayish-white vegetation measuring 0.5 cm × 0.5 cm was observed on the aortic valve. **(B)** Hematoxylin and eosin (H&E) staining at 10 × 10 magnification revealed a thrombotic vegetation, with bacterial clusters identified within the thrombus.

On day 3, the patient developed a low-grade fever accompanied by elevated inflammatory markers (Table 2). Intraoperative findings revealed the presence of vegetations on the aortic valve, prompting an escalation of antimicrobial therapy to include piperacillin-tazobactam (4.5 g administered via infusion pump every 6 h) and vancomycin (1 g administered via infusion pump every 12 h). This represents the second modification of the antibiotic treatment regimen. The resected vegetations were sent for histopathological examination, tissue bacterial culture, and capture-based mNGS (Figure 2). On day 4, the capture-based mNGS results of the valve vegetation (Figure 3) identified *S. cristatus* with 250,119 reads and a coverage rate of 54.56%. The patient is currently afebrile, and in conjunction with the capture-based mNGS results, the current anti-infective treatment program will continue, while closely monitoring the patient's temperature and blood parameters. Pathological findings of the valve vegetations on day 5 (Figure 4), indicated grayish-white vegetations on the aortic valve, with thrombotic vegetations and bacterial clusters observed microscopically in the valve vegetations, which were positively stained by Gram's stain. On day 8, tissue bacterial culture confirmed the presence of *S. cristatus*.

**Table 2:** Trend plot of inflammatory markers.

Date	Inflammatory markers	
	PCT (0.0–0.060 ng/ml)	IL-6 (0.0–7.0 pg/ml)
d0	0.561	124.0
d1	0.464	65.2
d2	0.434	13.9
d3	0.481	73.7
d4	0.504	34.0

ICU admission is used as the day 0 reference point. IL-6, interleukin-6; PCT, procalcitonin.

On day 6, the patient in stable condition was transferred from the ICU to the general cardiac surgery ward for continued management. The patient was discharged on day 26 following an uncomplicated recovery. Three weeks post-discharge, the patient presented for a routine follow-up evaluation. On physical examination, breath sounds were clear bilaterally without adventitious sounds such as crackles or wheezes. Cardiac auscultation revealed regular rate and rhythm with no appreciable murmurs over the valve areas. Laboratory studies including a complete blood count and serum biochemistry were within normal limits. Transthoracic echocardiography demonstrated normal prosthetic valve (bioprosthetic valves) function with laminar flow through the graft conduit.

## Discussion and conclusion

*S. cristatus* is a gram-positive, catalase-negative streptococcus originally isolated from the human oral cavity, specifically from teeth and gingiva, and is known for its cariogenic properties (11). This organism was first definitively classified through phenotypic identification techniques in 1991 and is typically regarded as a commensal organism that is generally non-pathogenic (12). However, recent studies have indicated that *S. cristatus* can be implicated in a variety of infections, including periodontal abscesses, periodontitis, endocarditis, osteomyelitis, endophthalmitis, and septic arthritis (13–17). Notably, recent reports have highlighted that *S. cristatus* can lead to severe infective endocarditis with complications, even in individuals who do not have underlying health conditions (16–18). The modified Duke criteria expands the list of microorganisms considered “typical pathogens” (1). This list of typical pathogens includes *Staphylococcus aureus*, *Staphylococcus lugdunensis*, *Enterococcus faecalis*, all *Streptococcus species* (except *Streptococcus pneumoniae* and *Streptococcus pyogenes*), *Granulicatella* spp., *Abiotrophia* spp., *Gemella* spp., and microorganisms from the HACEK group [*Haemophilus* species, *Aggregatibacter actinomycetemcomitans*, *Cardiobacterium hominis*, *Eikenella corrodens*, and *Kingella kingae*].

Infective endocarditis can rapidly progress to heart failure, infective embolism, and even death, making early and accurate diagnosis crucial (19). In this case, the patient was admitted to the hospital with acute heart failure and myocardial infarction resulting from valvular regurgitation. Following comprehensive investigations upon admission, clinical suspicion of infective endocarditis was raised, leading to surgical treatment after a cardiac surgery consultation to evaluate surgical indications. Possible reasons for negative blood cultures in infective endocarditis include the administration of antibiotic therapy prior to pathogen examination, harsh culture conditions, or the slow growth rate of pathogenic bacteria (20). However, identifying pathogenic organisms through blood cultures and associated infected tissue cultures is vital for guiding antimicrobial treatment and subsequent management. Ana Sofia Silva et al. (17) reported a case of native valve infective endocarditis caused by *S. cristatus*. The case report describes a patient presenting with a two-week history of unexplained fever. The diagnosis of *S. cristatus* endocarditis was established based on positive S. cristatus blood cultures, echocardiographic identification of valvular vegetations, and the complication of splenic embolism (17). In this case, the patient exhibited negative preoperative blood cultures, and the pathogen could not be identified early through blood cultures, potentially due to the administration of empiric anti-infective therapy prior to pathogen identification.

The diagnosis of pathogens in patients with blood culture-negative endocarditis often necessitates the use of additional tests beyond blood culture, such as serologic tests or PCR (5). More than 20% of cases of blood culture-negative endocarditis still lack effective tests for pathogen identification (21). Renate Ziegler et al. (22) reported that 61.1% of IE cases with negative blood cultures identified microorganisms by 16S rDNA and only 19.4% by heart valve culture. The spectrum of causative agents of infective endocarditis has changed significantly over the past few decades. Concurrently, the emergence of new diagnostic techniques and advancements in cardiac surgical methods have led to the 2023 version of the Duke-ISCVID IE diagnostic criteria, which offer clinicians a comprehensive and systematic approach to diagnosing infective endocarditis (1). Notably, recent years have seen NGS technology emerge as a powerful tool for accurately detecting pathogen genomes and drug resistance genes, thereby providing a molecular foundation for precise diagnosis and informed postoperative antibiotic selection. This is expected to shorten treatment duration and mitigate the risk of drug resistance and recurrence (7, 8). Furthermore, optimizing postoperative antibiotic regimens through NGS technology can lower healthcare costs (e.g., by reducing the unnecessary use of broad-spectrum antibiotics), enhance patient survival, and advance the implementation of precision medicine in the field of infectious diseases.

In this case, the diagnosis of *S. cristatus* IE was ultimately confirmed through capture-based mNGS analysis of surgically resected valve vegetations. Capture-based mNGS provided etiologic diagnosis within 24 h, significantly faster than traditional valve cultures (results was reported after 5-day culture), suggesting that capture-based mNGS, as a newly applied pathogen detection technology in recent years, has obvious advantages in rapid and accurate diagnosis of pathogenic microorganisms, and is expected to become an important auxiliary means in addition to the conventional detection methods. Domingo Fernández Vecilla et al. (18) reported a case of *S. cristatus* endocarditis in a 72-year-old patient re-diagnosed after bioprosthetic valve replacement. Routine culture of the valvular tissue excised during the second valve replacement surgery yielded negative results. Subsequently, 16S ribosomal RNA gene sequencing was performed on the valvular tissue, resulting in a 638 bp gene sequence. Comparative analysis against the BLASTR database identified the sequence as *S. cristatus*. Currently, mNGS has been widely adopted in China for respiratory infection diagnostics (23). Its ability to detect a broad range of potential pathogens in clinical samples provides superior comprehensiveness compared to serological tests and 16S rRNA PCR (23). However, the application of this technology in IE remains largely exploratory. The capture-based mNGS employed in this case builds upon conventional mNGS library preparation by utilizing extensive probe panels to enrich pathogens, comprehensively covering bacteria, fungi, viruses, antimicrobial resistance genes, and virulence factors. This method directly captures pathogenic microorganisms along with their associated virulence and/or resistance genes from clinical specimens prior to high-throughput sequencing. Subsequent bioinformatics alignment against reference databases allows for precise microbial identification, providing critical evidence for clinical diagnosis and treatment. Compared to conventional mNGS, this approach achieves up to a 100-fold improvement in pathogen detection sensitivity while requiring only 1/20th of the standard sequencing volume (24, 25). Its cost-effectiveness underscores significant value in health economics (24, 25).

Patients with blood culture-negative endocarditis are generally treated empirically with anti-infective therapy after admission to the hospital for definitive diagnosis. Guidelines recommend that penicillin and second-generation cephalosporins remain the first choice, followed by vancomycin and linezolid (2, 26). In this case report, following the patient's admission to the ICU, the attending physician administered moxifloxacin for a 2-day course of empirical antibiotic therapy based on personal antimicrobial preference, failing to adhere to the recommended prophylactic antibiotic regimen outlined in the international IE guidelines. Postoperatively, antimicrobial therapy was transitioned to vancomycin plus piperacillin-tazobactam, in accordance with the guidelines and based on the patient's evolving clinical condition. Both the capture-based mNGS results from valve vegetations and the patient's clinical improvement confirmed the efficacy of this therapeutic adjustment. The patient in this case expressed satisfaction with the rapid and accurate identification of the causative pathogen of IE through capture-based mNGS and conveyed gratitude for the entire treatment process and outcome. At the 3-week postoperative follow-up, the patient was recovering well, with no reported complications.

As a single case report, its results are challenging to compare statistically with other methods, and its generalizability necessitates validation through future large-scale studies. The value of this case lies in providing proof of concept and insights into future research directions. Capture-based mNGS significantly enhances detection sensitivity and specificity by enriching pathogen nucleic acids using extensive probe panels. This approach is particularly advantageous for samples with high host background (e.g., blood) or low pathogen load, effectively overcoming the detection limitations of conventional mNGS while reducing sequencing costs. However, this method relies entirely on reference sequences of known pathogens for probe design, rendering it incapable of detecting novel pathogens or highly divergent strains. Furthermore, it compromises its ability to perform comprehensive microbial community analysis. Additionally, the technical workflow is complicated by the inclusion of a hybridization capture step and exhibits sequence capture bias.

In summary, our case report indicates that valvular capture-based mNGS is a relatively rapid and efficient diagnostic method for rapid pathogen clarification and precision of anti-infective medication in postoperative IE patients with persistent infections and negative blood cultures. However, given that valvular capture-based mNGS is not yet widely implemented in the context of IE, its diagnostic benefits and limitations warrant further investigation.

## Data Availability

The original contributions presented in the study are publicly available. This data can be found here: https://www.ncbi.nlm.nih.gov/bioproject/PRJNA1301542.
